# Impact of *Lactobacillus reuteri* Supplementation on Anti-*Helicobacter pylori* Levofloxacin-Based Second-Line Therapy

**DOI:** 10.1155/2012/740381

**Published:** 2012-05-29

**Authors:** Veronica Ojetti, Giovanni Bruno, Maria Elena Ainora, Giovanni Gigante, Gianluca Rizzo, Davide Roccarina, Antonio Gasbarrini

**Affiliations:** ^1^Departement of Internal Medicine, Catholic University of Rome, 00168 Rome, Italy; ^2^Departement of Surgery, Catholic University of Rome, 00168 Rome, Italy

## Abstract

*Introduction*. *Helicobacter pylori* eradication therapy has the potential burden of antibiotic-associated gastrointestinal (GI) side effects. The occurrence of side effects is among the major drawbacks of such regimens. GI manifestations may be related to alterations in the intestinal microflora. Probiotics can prevent or reduce antibiotic-associated side effects and have an inhibitory effect on *H. pylori*. *Methods*. To define the efficacy of *Lactobacillus reuteri* supplementation in *H. pylori* eradication and in preventing GI-associated side effects during a second-line levofloxacin triple therapy. 90 *H. pylori*-positive patients receive for 7 days a second-line triple therapy with esomeprazole, levofloxacin, and amoxicillin with *L. reuteri* for 14 days (group 1) and without probiotic supplementation (group 2). Each subject received a validated questionnaire to record symptoms everyday for 4 weeks from the start of therapy. *H. pylori* status and side effects were assessed 6 weeks after treatment. *Results*. The eradication rate was significantly influenced by probiotic supplementation with *L. reuteri* (group 1: 36/45, 80%; group 2: 28/45 62%; *P* < 0.05). The incidence of nausea and diarrhoea in group 1 was significantly lower than that in group 2. *Conclusion*. In *H. pylori*-positive subjects *L. reuteri* supplementation increases the eradication rate while reducing the incidence of the most common side effects associated with antibiotic therapy in second-line treatment.

## 1. Introduction

*Helicobacter pylori *(*H. pylori*), a microaerophilic, gram negative bacterium that colonises the mucous layer of the gastric epithelium, is the causative agent of type B gastritis, peptic ulcer, gastric cancer [1–3], and extradigestive diseases [4]. At least one-third of the world's population is infected with *H. pylori*. The standard treatment recommended for *H. pylori *eradication is a combination of proton-pump inhibitor (PPI) or ranitidine bismuth citrate, clarithromycin, and either amoxicillin or nitroimidazole. These regimens have been able to achieve eradication rates ranging from 65% to 90%; however they have the disadvantage of being expensive and cause side effects which require the withdrawal of therapy and antibiotic resistance can be developed [5].

According to Maastricht III consensus the second-line treatment should be bismuth-based quadruple therapy (if available), PPI plus amoxicillin, tetracycline or metronidazole [6]. Our group report in 2009 a levofloxacin-based triple therapy as a valid alternative [7].

2 papers have shown also the superiority of levofloxacin triple over bismuth quadruple therapy [8, 9].

As regards antibiotic resistance rate a high resistance versus metronidazole and clarithromycin was reported in our country.

An interesting paper by Romano et al. report a high eradication rate with levofloxacin versus clarithromycin and the success depends at least in part on the very low prevalence in levofloxacin-resistant *H. pylori* strains in our population (3%) [10].

Antibiotic-associated gastrointestinal side effects such as diarrhoea, nausea, vomiting, bloating, and abdominal pain can represent a serious drawback to anti-*H. pylori *therapies. These manifestations have been related to quantitative and qualitative changes in the intestinal microflora because of unabsorbed or secreted antibiotics in the intestinal content, with a resulting reduction in normal saprophytic flora, overgrowth and persistence of potentially pathogenic antibiotic-resistant indigenous strains [11].

At present, treatment failure is a significant problem in clinical practice, and the possibility to use simpler eradication schemes or new drugs should be regarded as the most promising way to improve the efficacy of eradication therapy. Some papers showed that the use of probiotics during the first-line *H. pylori *therapy improved the patients compliance and reduced gastrointestinal symptoms [12–14].

A probiotic is defined as a living microbial species that, on administration, can have a positive effect on bowel microecology with improved health conditions. At present, the most studied probiotics are lactic acid-producing bacteria, particularly *Lactobacillus *[15, 16]. Probiotics have been proven to be useful in the treatment of several gastrointestinal diseases such as acute infectious diarrhoea or pouchitis [17, 18]. Moreover, as shown in several studies, probiotics also show a direct antimicrobial effect [19]. In particular, probiotics may compete directly with *H. pylori*, possibly through the inhibition of adherence, as well as by producing metabolites and antimicrobial molecules [20].

On this basis, the Maastricht 2–2000 Consensus Report speculated on the role of probiotic supplementation in the treatment of *H. pylori *infection [21].

The implementation of standard anti-*H. pylori *regimens with probiotics could be advisable, as they are able to improve the patient's compliance by reducing antibiotic-associated adverse events, thus increasing the number of patients completing eradication therapy, resulting in improved eradication rate [22–24]. However, the number of patients enrolled in these trials was too small to achieve statistically conclusive results.

*Lactobacillus reuteri (L. reuteri) *in one of the most interesting lactobacillus, with some stimulating properties; in particular, it is antibiotic resistant, improves the immune response in the gastrointestinal tract, has a therapeutic effect in acute diarrhoea, reduces the incidence of antibiotic-associated side effects, and inhibits *H. pylori *in vitro and in vivo [25–28]. A recent study reports that a first-line therapy with 4-week *L. reuteri *supplementation is effective in reducing *H. pylori *bacterial load in humans and theoretically may help to control gastric inflammation [29]. 

The aim of our study was to define the efficacy of *L. reuteri *supplementation in *H. pylori *eradication and in preventing associated gastrointestinal side effects during anti-*H. pylori *infection second-line levofloxacin triple therapy.

## 2. Methods

### 2.1. Patients

The study was a single-centre, prospective, randomised, controlled study performed at the Gastroenterology and Internal Medicine Departments of Gemelli Hospital of Rome, Italy.

All patients are Caucasian and came from the same geographic area.

Ninety consecutive *H. pylori*-positive patients were enrolled from November 2007 to June 2008. Patients were considered eligible to enter the study if they were between 18 and 65 years old, affected by gastric *H. pylori *infection as confirmed by a 13C-urea breath test, submitted to a previous unsuccessful anti-*H. pylori *antibiotic treatment. Exclusion criteria were recent (within the previous 3 months) use of antimicrobial agents, bismuth compounds, PPI and H2 receptor antagonists, laxatives, antidiarrheal, other probiotic preparations, alcohol, or drug abuse. Patients with major concomitant diseases including psychiatric disorders and pregnant or lactating women were also excluded from the study. All patients signed a written informed consent. The study was approved by our Ethical Committee.

### 2.2. Treatment

Using a permuted block randomization (1 : 1), 90 patients were assigned to one of the following parallel groups.

- 45 patients (32 males/13 females, mean age 41.5 ± 11.7) were randomly assigned to receive a triple therapy based on esomeprazole 20 mg bid, levofloxacin 500 mg bid, and amoxicillin 1 gr bid for 7 days plus *L. reuteri *(1 × 10^8^, CFU) (Reuflor Italchimici Pomezia, Italy) t.i.d for 14 days, during eradication therapy and 1 week thereafter.
- 45 patients (28 males/17 females, mean age 43.1 ± 13.3) were randomly assigned to receive the same triple therapy without probiotics.

### 2.3. Side Effects

Each patient was required to complete a validated daily diary for 2 weeks, starting from the first day of eradication treatment. The diary contains a questionnaire (slightly modified from De Boer et al.)[30] evaluating the onset, intensity, and frequency of gastrointestinal side effects: taste disturbance, epigastric pain, constipation, skin rash, nausea, vomiting, abdominal pain, bloating, loss of appetite, and diarrhoea. Symptom intensity was rated using a scale, where 0, 1, 2, and 3 corresponded to absent, mild, moderate and severe symptoms, respectively. An overall judgment of tolerability was assessed by the patient at the end of both the first and second weeks of treatment. Treatment compliance was evaluated by counting the vials returned by the subject (patients who returned <80% of empty vials were not included in the per protocol population (PP) analysis). Patients were adequately informed and motivated to therapy, and strictly.

### 2.4. Eradication of Helicobacter pylori

*H. pylori *status was controlled with the 13C urea breath test performed with citric acid and 75 mg of 13C urea, with the eradication control test being performed not before 6 weeks after the end of therapy [31, 32]. A delta value higher than 3.5 units was considered the cut-off for positivity.

### 2.5. Statistical Analysis

To evaluate *H. pylori *eradication three variables, previously dichotomised, were analysed: *L. reuteri *supplementation (Y versus N), sex (males versus females), and age (age < median age versus age ≥ median age). A univariate analysis was performed with the chi-squared test. The significant cut-off was set at *P* < 0.05. Significant parameters with a *P* value less than 0.25 at univariate analysis were entered in a multivariate logistic regression model to identify independent predictors of *H. pylori* eradication. Odds ratio (OR) to achieve *H. pylori *eradication with 95% confidence intervals (CI) was calculated.

The statistical analysis of side effects was performed with the chi-square univariate analysis. All variables were dichotomised into two group: symptoms Y versus symptoms N and moderate-severe symptoms versus negligible or not referred symptoms. The significant cut-off was *P* < 0.05.

All analyses were performed using SYSTAT 12.0 for Windows.

### 2.6. End Point

The primary end point of the study was to compare the eradication rate achieved with the triple therapy with or without *L. reuteri *supplementation.

The secondary end point were the patients compliance and the occurrence of side effects in the two groups of different treatment.

## 3. Results

All patients completed the study.

Forty-five patients were treated with *L. reuteri *supplementation (group 1) and 45 patients were treated without probiotic supplementation (group 2).

The per protocol and “intention to treat” analyses were shown to be the same in our study, because of the absence of drop out events. The overall patients compliance to both eradication schemes was good, with all patients completing the prescribed therapy.

A significantly higher eradication rate was achieved in group 1 with 80% eradication rate (36/45), compared to 60% (27/45) in group 2 (*P*: 0.038). Age (*P*: 0.782) and sex (*P*: 0.329) had no significant impact on *H. pylori *eradication rate (Table 1). *L. reuteri*, age and sex were evaluated in a multivariate model of statistical analysis and we found that *L. reuteri *supplementation was the only predicting factor in *H. pylori *eradication (*P*: 0.026; odds ratio: 3.055; confidence interval: 1.146–8.150) (Table 2).

As regards the analysed side effects, taste distortion was referred by 6 patients (13.3%) of group 1 and by 8 patients (17.8%) of group 2 (*P*: 0.561); epigastric pain was reported by 5 patients (11.1%) of group 1 and 4 patients (8.9%) of group 2 (*P*: 0.725); constipation was reported by 8 patients (17.8%) of group 1 and 11 patients (24.4%) of group 2 (*P*: 0.438); skin rash was observed in 4 patients (8.9%) of both groups (*P*: 1.000). No patients referred moderate or severe taste disturbance, epigastric pain, constipation and skin rash of both groups.

Thirty patients (66.7%) of group 1 reported nausea, 19 (42.2%) of them of moderate-severe intensity, while all patients (100%) of group 2 referred moderate-severe nausea (*P* < 0.001 both in absolute terms and for moderate-severe symptoms). No significant difference was reported for vomiting that was referred by 17 patients (37.8%) of group 1 and 15 patients (33.3%) of group 2 (*P*: 0.660), with a moderate severe score by 3 patients (6.7%) of both groups (*P*: 1.000). Twenty-nine patients (64.4%) of group 1 and 31 patients (68.9%) of group 2 referred abdominal pain (*P*: 0.655); this symptom was reported as moderate severe by 6 patients (13.3%) of group 1 and by 12 patients (26.7%) of group 2 (*P*: 0.114). Bloating was reported by 35 patients (77.8%) of group 1 and 37 patients (82.2%) of group 2 (*P*: 0.598), which was of moderate-severe intensity by 12 patients (26.7%) of group 1 and 8 patients (17.8%) of group 2, respectively (*P*: 0.310). 36 patients (80%) of group 1 and 33 patients (73.3%) of group 2 referred loss of appetite (*P*: 0.455), which was of moderate-severe intensity in 11 patients (24.4%) and 15 patients (33.3%), respectively, (*P*: 0.352). Diarrhoea was reported by 10 (22.2%) patients of group 1 and 26 (57.7%) of group 2 (*P*: 0.004); it was moderate-severe in 4 patients (40.0%) and in 15 patients (57.6%), respectively (*P*: 0.001) (Table 3).

## 4. Discussion

In the present randomised controlled study we have shown that patients treated with *L. reuteri *during a levofloxacin-based second-line *H. pylori *therapy experienced a lower incidence of nausea and diarrhoea compared to subjects without probiotic supplementation and with a higher eradication rate. Probiotics may act in a different way: by direct competition with *H. pylori *or by improving the patients compliance to therapy reducing the incidence of antibiotic-associated side effects [33–35]. The direct effect against *H. pylori *is supported only by animal and in vitro studies while several others have confirmed that probiotics indirectly improve eradication rate with reduced incidence of side effects and improved patients compliance [36–38].

Currently, the best studied probiotics are the lactic acid bacteria, in particular *Lactobacillus* and *Bifidobacterium* [39–43]. In our study we have used *L. reuteri *ATCC 55730 because previous clinical trials have shown that its administration is safe in both adults and children, reducing the incidence and severity of gastrointestinal side effects; moreover it is bile and acid resistant, adheres to the mucosa and to enterocytes and inhibits *H. pylori *growth in vitro and vivo [44, 45].

Previous studies reported that *L. reuteri *has the cell surface protein that inhibits in vitro the binding of *H. pylori* to receptor glycolipids (asialo-GM1 and sulfatide) [19]. To confirm this data Canducci et al. [26] have recently published a randomised placebo-controlled study and have shown an inhibitory effect of *L. reuteri *on *H. pylori *growth with a significant decrease in both 13C-UBT and *H. pylori*. Thus, *L. reuteri *seems to exert a beneficial effect during *H. pylori *infection resulting in a reduction of bacterial load and gastric inflammation. In the literature, many clinical trials are reported on the use of single or multiple probiotic strains administered for *H. pylori *treatment. Unfortunately, it is hard to compare these trials because of different randomisation, probiotic administration, doses, and concomitant therapy.

The second aim of our study was to assess whether *L. reuteri *could be of help in ameliorating symptoms during *H. pylori *triple therapy. We have shown that patients receiving the probiotic experienced a significant improvement of some gastrointestinal symptoms compared to those without probiotic supplementation.

In particular symptoms with a lower incidence in group 1 treated with *L. reuteri *were diarrhoea and nausea. Previous studies have shown that oral probiotic treatments during first-line anti-*H. pylori* regimens were able to reduce the incidence of diarrhoea, nausea and taste disturbance. It is well known that antibiotic-associated side effects are common and are usually the first cause of therapy withdrawal. In fact, antibacterial drugs, can alter the equilibrium between bacterial concentration and colonic mucosal cells, causing a prevalence of pathogens over the normal microflora. Probiotic supplementation may partially restore the intestinal physiological microecology [46–48].

A Cochrane analysis showed that antibiotics alter the microbial balance within the gastrointestinal tract and probiotics [49]. In particular, *Lactobacillus spp.* and *Saccharomyces boulardii* can prevent antibiotic-associated diarrhoea by restoring the gut microflora [42–51].

A recently published paper has shown that *L. reuteri *is effective in reducing gastrointestinal symptoms during *H. pylori *eradication therapy in children [29–33].

A meta-analysis on the effects of probiotic supplementation on eradication rates and adverse events during *H. pylori *eradication therapy suggests that probiotics are effective in increasing the eradication rate and can be considered helpful for patients with eradication failure [52]. However, there are only two trials which confirm this conclusion: this is the reason why to confirm this result; we have designed a randomised controlled trial in a large population.

A major drawback of our study is the lack of a double-blind controlled design; so the difference in side effects needs to be judged with caution.

Our study is the first that has evaluated the administration of *L. reuteri *in levofloxacin second-line therapy. It is well known that a second-line therapy results in success rates from less than 60% to 90% with the most relevant determinant of success being microbial sensitivity and patients compliance.

In summary, we confirm that *L. reuteri *supplementation during a second-line *H. pylori *therapy is recommended first of all for a better eradication rate and second for reduced gastrointestinal side effects. Although this pilot study opens new areas of investigation, further studies on a larger number of patients are required to define its real clinical application.

## Figures and Tables

**Table 1:** HP-eradication: univariate analysis.

Independent variables	Subgroups analysed	HP eradication rate	*P* value
*L. reuteri* supplementation	Y	36 (80%)	*0.038 *
	N	27 (60%)	
Age	<42 y	33 (73.3%)	0.782
	≥42 y	30 (66.7%)	
Sex	Male	40 (88.9%)	0.329
	Female	23 (51.1%)	

**Table 2:** HP-eradication: multivariate analysis.

Independent variables	*P* value	Odds ratio	95% Confidence interval
*L. reuteri* supplementation	*0.026*	*3.055*	* 1.146–8.150 *
Age	0.434	1.471	0.559–3.869
Sex	0.195	0.497	0.172–1.430

**Table 3:** HP-eradication: univariate analysis of symptom regression.

Symptoms	*L. reuteri* group	No *L. reuteri* group	*P* value
Nausea	32 (66.7%)	45 (100%)	*** <0.001 ***
Moderate-severe nausea	19 (42.2%)	45 (100%)	*** <0.001 ***
Abdominal pain	29 (64.4%)	31 (68.9%)	0.655
Moderate-severe abdominal pain	6 (13.3%)	12 (26.7%)	0.114
Diarrhoea	10 (22.2%)	26 (57.7%)	*** <0.004 ***
Moderate-severe diarrhoea	4 (10%)	15 (57.6%)	*** <0.001 ***
Bloating	35 (77.7%)	37 (82.2%)	0.598
Moderate-severe bloating	12 (26.7%)	8 (17.8%)	0.310
Loss of appetite	36 (80%)	33 (73.3%)	0.455
Moderate-severe loss of appetite	11 (24.4%)	15 (33.3%)	0.352
Vomiting	17 (37.8%)	15 (33.3%)	0.660
Moderate-severe vomiting	3 (6.7%)	3 (6.7%)	1.000
Epigastric pain	5 (11.1%)	4 (8.9%)	0.725
Taste disturbance	6 (13.3%)	8 (17.8%)	0.561
Skin rash	4 (8.9%)	4 (8.9%)	1.000
Constipation	8 (17.8%)	11 (24.4%)	0.438
